# Significance of NS5B Substitutions in Genotype 1b Hepatitis C Virus Evaluated by Bioinformatics Analysis

**DOI:** 10.1038/s41598-018-27291-7

**Published:** 2018-06-11

**Authors:** Yoshihito Uchida, Shugo Nakamura, Jun-Ichi Kouyama, Kayoko Naiki, Daisuke Motoya, Kayoko Sugawara, Mie Inao, Yukinori Imai, Nobuaki Nakayama, Tomoaki Tomiya, Charlotte Hedskog, Diana Brainard, Hongmei Mo, Satoshi Mochida

**Affiliations:** 1 Department of Gastroenterology & Hepatology, Faculty of Medicine, Saitama Medical University, Saitama, Japan; 2 Faculty of Information Networking for Innovation and Design, Toyo University, Toyo, Japan; 3 Gilead Sciences, Inc., Foster City, California USA

## Abstract

To evaluate the effects of HCV NS5B amino acid substitutions on treatment outcome in Ledipasvir (LDV)/Sofosbuvir (SOF) for Japanese patients with genotype 1b HCV infection, NS5B sequences were examined in i) seven patients experiencing virologic failure after LDV/SOF in real-world practice, ii) 109 SOF-naïve patients, iii) 165 patients enrolled in Phase-3 LDV/SOF trial. A218S and C316N were detected in all patients with viral relapse; the percentages of these substitutions in SOF-naïve patients were 64.2% and 55.0%, respectively. Genotype 1b HCV strains with NS5B-C316N mutation were located in the leaves different from those in which HCV strains without such substitutions were present on the phylogenetic tree. Structural modeling revealed that amino acid 218 was located on the surface of the NTP tunnel. Free energy analysis based on molecular dynamics simulations demonstrated that the free energy required to pass through the tunnel was larger for triphosphate SOF than for UTP in NS5B polymerase carrying A218S, but not in wild-type. However, no susceptibility change was observed for these substitutions to SOF in replicon assay. Furthermore, the SVR rate was 100% in patients enrolled the Phase-3 trial. In conclusion, NS5B A218S and C316N were detected in all patients who relapsed following LDV/SOF in real-world practice. These substitutions did not impact the overall SVR rate after LDV/SOF, however, further studies are needed to elucidate the impact of these substitutions.

## Introduction

Combination therapies using 2 or 3 direct-acting antiviral agents (DAAs) have improved sustained viral response (SVR) rates in patients with genotype 1b HCV. DAAs are classified into 3 categories: nonstructural (NS) 3/4 A protease inhibitors, NS5A inhibitors and NS5B polymerase inhibitors. In Japan, dual oral therapy with daclatasvir (DCV), an NS5A inhibitor, and asunaprevir (ASV), a second-generation NS3/4 A protease inhibitor, was approved for patients with genotype 1b HCV as the first interferon-free regimen in July 2014^1^. In DAA therapies including NS5A inhibitors, a baseline resistance-associated substitution (RAS), especially the NS5A-Y93H mutation, was shown to be associated with virologic failure^1^. Thus, we developed a simple assay to quantify the percentages of HCV-RNA levels of NS5A-Y93H mutant HCV strains and NS5A-Y93 wild-type HCV strains relative to the total HCV-RNA levels using cycling-probe real-time polymerase chain reaction (PCR)^2^ and established a diagnostic system in combination with direct sequencing to evaluate NS5A-RAS, including the NS5A-L31M/V mutation^3^. Also, Yoshimi *et al*. established an assay system for NS5A-RAS using the INVADER method^4^. These diagnostic systems contributed to an improvement in the therapeutic efficacies of dual oral therapies with DCV plus ASV in real-world practice through the exclusion of patients with HCV strains carrying NS5A-RAS from the potential candidates for treatment^5^.

Another type of DAA, known as NS5B polymerase inhibitors, is classified into nucleotide and non-nucleotide inhibitors; sofosbuvir (SOF)^6^ belongs to the former classification, while beclabuvir (BEC)^7^ and dasabuvir (DSB)^8^ belong to the latter. In Japan, SOF was approved for clinical use for patients with genotype 1b HCV in July 2015 as a combination tablet with ledipasvir (LDV), an NS5A inhibitor. The therapeutic efficacy of DAAs, including SOF-based treatments, was shown to be excellent;^9,10^ especially in a Japanese trial^10^, all the patients with genotype 1 HCV who received LDV/SOF without ribavirin (RBV) achieved an SVR. In real-world practice, however, virologic failure can occur in patients with genotype 1b HCV even among those without previous DAA therapies including NS5A inhibitors^11^. For SOF, NS5B-S282T confers resistance to SOF with an EC_50_ value relative to that of NS5B-S282 wild-type HCV strains of 9.5^12^. Also, Donaldson *et al* reported that a baseline NS5B-C316N mutation was potentially associated with a reduced response to SOF in patients with genotype 1b HCV based on bioinformatics characterization^13^, whereas HCV strains carrying this mutation showed no resistance against SOF in an *in vitro* replicon system^14^.

Bioinformatics approach based on structural modeling and molecular dynamics simulations, was established by Karplus, Levitt and Warshel in 1976 as a multiscale model for analyzing complicated chemical reactions^15,16^. In current science, *in silico* assay is applied to verify the interaction, in which *in vitro* and *in vivo* experiments failed to clarify the mechanisms^17,18^. Molecular dynamics simulation is also used to sample conformations of molecules for free energy calculations and has been applied to various analyses such as protein-protein interactions, protein ligand interactions, protein folding, and so on^19–26^.

Thus, in the present study, we examined prevalence of HCV NS5B amino acid substitutions in patients failing treatment with LDV/SOF, and evaluated the impact of these substitutions at baseline on response to LDV/SOF treatment in patients with HCV genotype 1b infection in Japan. Moreover, the significance of these substitutions were assessed *in silico* by bioinformatics analysis as well as *in vitro* by replicon system.

## Results

### Amino Acid Substitutions in the NS5A and NS5B Region of Genotype 1b HCV in Patients Experiencing Virologic Failure after LDV/SOF Therapy

**Figure 1:**
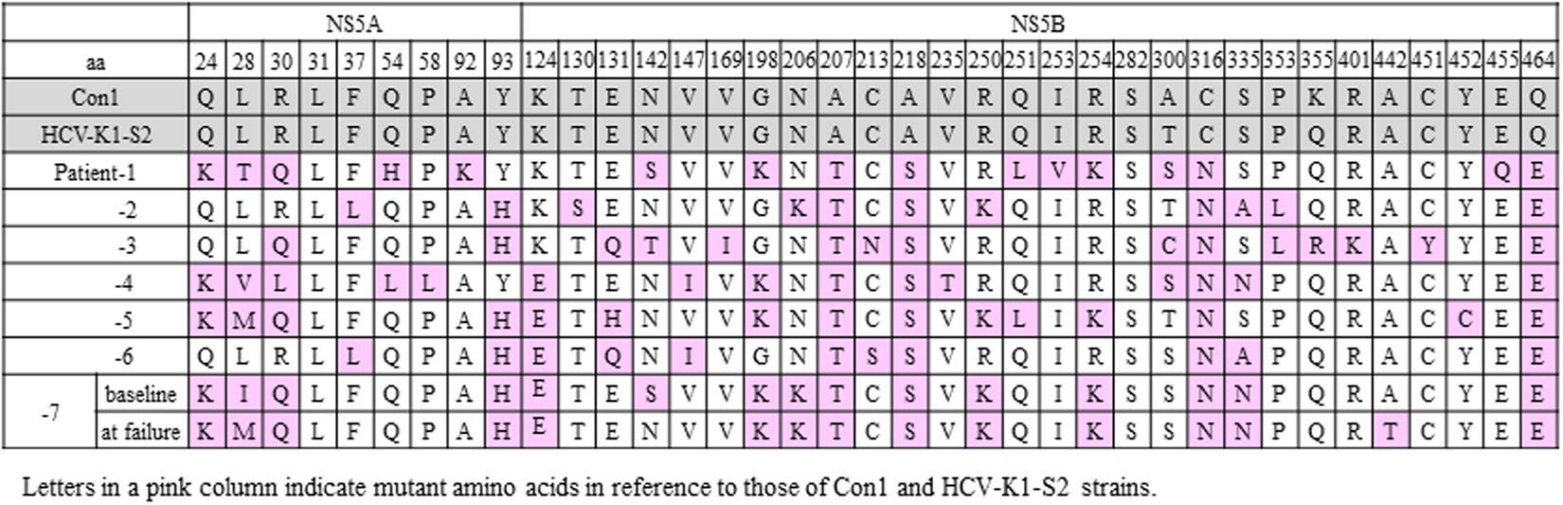
Amino Acid Mutations in the NS5A and NS5B Region of HCV Strains Obtained at Virologic Failure in 7 Patients Receiving Ledipasvir (LDV) and Sofosbuvir (SOF).

Amino acid substitutions in the NS5A and NS5B regions were evaluated in HCV sequences obtained from the 7 patients who experienced virologic relapse after LDV/SOF treatment (6 patients referred to our hospital after LDV/SOF failure and 1 patient of the 92 treated with LDV/SOF in our clinic). As shown in Fig. 1, NS5A-Y93H was found in 5 patients including 2 patients with HCV strains harboring NS5A-L28M/R30Q/Y93H mutations and NS5A-A92K in 1 patient, while NS5A-L31 was absent in all patients. On the other hand, in NS5B region, several amino acid substitutions at various sites were identified to be different from 1b reference virus. Four amino acid substitutions were found in all 7 patients: A207T, A218S, C316N and Q464E in the NS5B region. The NS5B-S282T was absent in all 7 patients.

Among these 7 patients, the baseline serum sample was available only in one patient (Patient-7), who had previously experienced virologic failure after DCV plus ASV therapy. In this patient, triple RAS in the NS5A region (L28I, R30Q, and Y93H) was detected at the baseline of LDV/SOF therapy. The quadruple amino acid substitutions in the NS5B region (A207T, A218S, C316N and Q464E) were also observed at baseline in this patient.

### Amino Acid Substitutions in the NS5B Region of Genotype 1b HCV in 109 SOF-Naïve Patients

**Figure 2:**
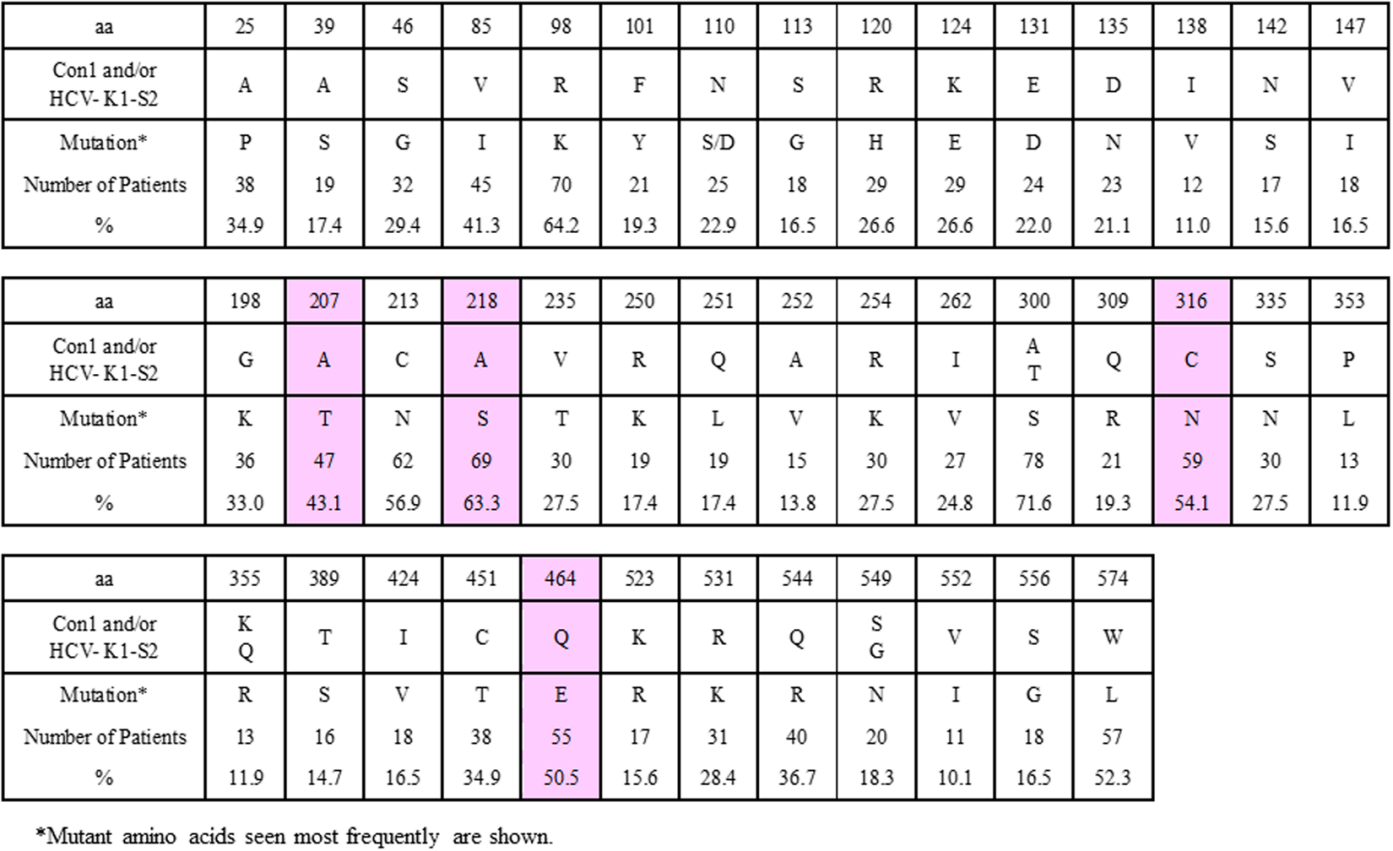
Frequencies of Amino Acid Mutations in the NS5B Regions of Genotype 1b HCV among 109 Patients without Previous Sofosbuvir Administration.

**Figure 3:**
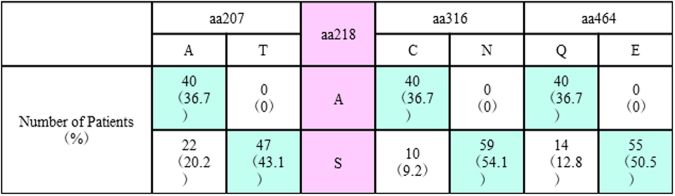
Linkage Disequilibrium between Phenotype of aa218 and Phenotypes of aa207, aa316, aa464 in the NS5B Region of Genotype 1b HCV in 109 Sofosbuvir-Naïve Patients.

**Figure 4:**
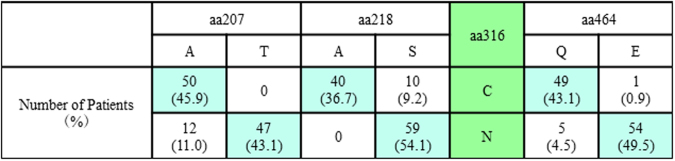
Linkage Disequilibrium between Phenotype of aa316 and Phenotypes of aa207, aa318, aa464 in the NS5B Region of Genotype 1b HCV in 109 Sofosbuvir-Naïve Patients.

**Figure 5:**
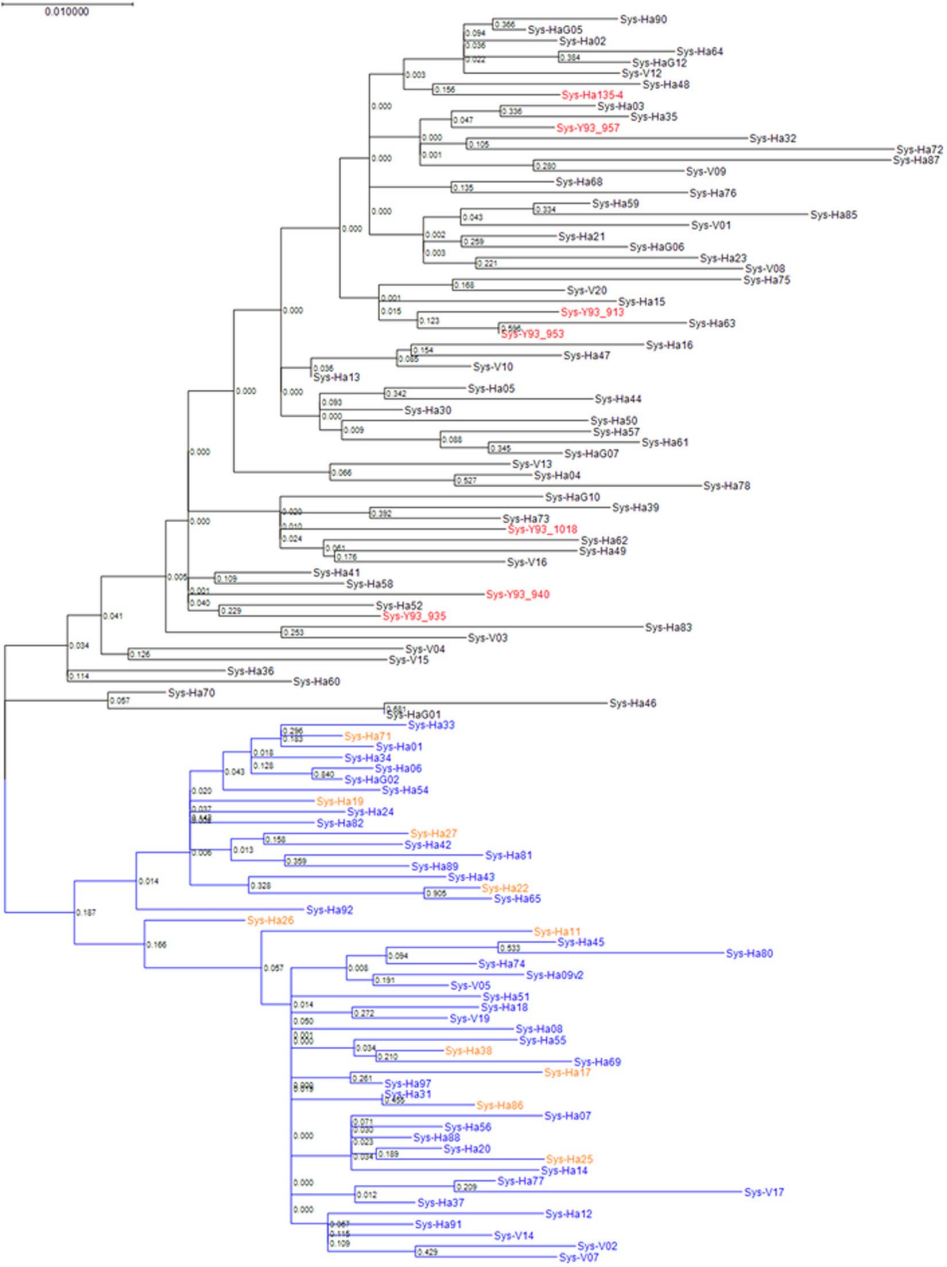
Phylogenetic Analysis in the NS5B region of Genotype 1b HCV. The genetic distance was estimated using Kimura 2 parameters, and a phylogenetic tree was constructed based on the nucleotide sequences in the NS5B region using the maximum likelihood method adapted with best fit model (Kimura 2-parameter plus Gamma distributed with Invariant sites). The bootstrap values are indicated at each tree root. Sequences with A218 and C316 wild-type (n = 40), A218S mutant and C316 wild-type (n = 10), and A218S and C316N mutant HCV strains (n = 59) observed in 109 sofosbuvir (SOF)-naïve patients are shown in blue, orange and black, respectively. Also, HCV strains (n = 7) detected in patients experiencing virologic failure after SOF-based therapy are shown in red.

To further understand the pre-treatment prevalence of these NS5B amino acid substitutions in genotype 1b Japanese patients especially in our institutes, baseline samples from 109 SOF-naïve patients were assessed. The demographic and clinical features of the patients are shown in Table 1 Amino acid substitutions that differed from genotype 1b Con1 and/or HCV-K1-S2 reference strains with a prevalence of >10% among SOF-naïve patients are shown in Fig. 2. A207T, A218S, C316N and Q464E substitutions were found in 47 (43.1%), 69 (63.3%), 59 (54.1%) and 55 patients (50.5%), respectively, and linkage disequilibrium existed among these mutations; when aa218 exhibited the wild-type phenotype (A218), the other 3 amino acids also exhibited wild-type phenotypes (A207, C316 and Q464) (Fig. 3), while aa316 exhibited a mutant phenotype (C316N) and aa218 exhibited a mutant phenotype (A218S) (Fig. 4). Consequently, patients were classified into 3 groups**:** 40 patients (36.7%) with HCV harboring A207, A218, C316 and Q464 wild**-**type in the NS5B region, 47 patients (43.1%) with HCV harboring A207T, A218S, C316N and Q464E, and 22 patients (20.2%) with HCV harboring A218S with wild-type phenotypes at either aa207, aa316, or aa464. Phylogenetic trees based on the 115 amino acid sequences in the NS5B region are shown in Fig. 5. The systematic bias by with and without the NS5B-C316N substitutions is indicated by the localization of blue/orange and black leaves in two separate domains of the tree, and HCV strains with NS5B-C316N mutation were located in leaves different from those in which HCV strains without such mutations were present. Moreover, HCV strains obtained at virologic failure in 7 patients receiving LDV/SOF therapy were assigned to the C316N-mutation-positive leaves without formation of sub-clusters. Out of these 109 SOF-naïve patients, 92 patients received LDV/SOF, and all except one achieved SVR12 and the overall SVR12 rate was 98.9% (91/92). SVR12 rates were 100% (34/34) in patients with HCV virus harboring A207, A218, C316 and Q464 wild**-**type phenotypes, 97.2% (35/36) in those with HCV virus harboring A207T, A218S, C316N and Q464E substitutions.

**Table 1:** The Demographic and Clinical Features of 109 SOF-naïve Patients Subjected to Analysis for the NS5B Region of HCV.

	All patients (n = 109)	C316 (n = 50)	C316N (n = 59)	P values
Age (years old)^†^	67 (27–87)	67 (41–87)	67 (27–85)	0.749^a^
Men / Women^††^	39 (35.8) / 70 (64.2)	21 (42.0) / 29 (58.0)	18 (30.5) / 41 (69.5))	0.234^b^
**Previous Therapy** ^**††**^				
Naïve	69 (63.3)	28 (56.0)	41 (69.5)	0.101
IFN or Peg-IFN monotherapy	6 (5.5)	2 (4.0)	4 (6.8)	
Peg-IFN + RBV	21 (19.3)	14 (28.0)	7 (11.9)	
TVR/SMV/VAN + Peg-IFN +RBV	11 (10.1)	6 (12.0)	5 (8.5)	
DCV + ASV	2 (1.8)	0 (0)	2 (3.4)	
SOF/LDV	0 (0)	0 (0)	0 (0)	
Previous IFN/Peg-IFN^††^	38 (34.9)	22 (44.0)	16 (27.1)	0.073^b^
Previous RBV^††^	32 (29.4)	20 (40.0)	12 (20.3)	0.034^b^
Previous NS3/4A-PI^††^	13 (11.9)	6 (12.0)	7 (11.9)	1.000^b^
Previous NS5A-I^††^	2 (1.8)	0 (0)	2 (3.4)	0.499^b^
Previous therapies for HCC^††^	11 (10.2%)	3 (6.0)	8 (13.6)	0.221^b^
Hemoglobin (g/dL)^†^	13.9 (9.0–17.7)	13.9 (9.4–17.2)	13.7 (9.0–17.7)	0.874^a^
Platelets (10^3^/mm^3^)^†^	156 (46–374)	146 (46–374)	165 (53–350)	0.196^a^
Albumin (g/dL)^†^	4.1 (2.9–4.8)	4.1 (3.4–4.7)	4.1 (2.9–4.8)	0.270^a^
AST (U/L)^†^	40 (15–135)	40 (15–135)	36 (18–127)	0.440^a^
ALT (U/L)^†^	36 (10–167)	36 (10–167)	63 (34–119)	0.465^a^
α-fetoprotein (ng/mL)^†^	4.5 (2.0–57.1)	4.6 (2.0–57.1)	4.0 (2.0–21.6)	0.297^a^
HCV-RNA (Log IU/mL)^†^	6.2 (4.0–7.1)	6.1 (4.0–7.1)	6.4 (4.1–7.1)	0.208^a^
FIB4 index^†^	2.97 (0.36–17.25)	3.05 (0.82–14.71)	2.80 (0.36–17.25)	0.444^a^
<3.25^††^	60 (55.0)	27 (54.0)	33 (55.9)	0.849^b^
≥3.25^††^	49 (45.0)	23 (46.0)	26 (44.1)	

^†^Medium value (range). ^††^Number of patient (peacentages). ^a^Mann-Whitney *U*-test, ^b^Fisher’s exact test. ^c^Chi-squared test.

IFN, interferon; Peg-IFN, pegylated IFN; RBV, ribavirin; TVR, telaprevir; SMV, simeprevir; VAN, vaniprevir; DCV, daclatasvir; ASV, asnaprevir; SOF, sofosbuvir; HCC, hepatocellular carcinoma; AST, aspartate aminotransferase; ALT, alanine aminotransferase.

Among 165 genotype 1b patients who received LDV/SOF as a part of a phase 3b clinical trial in Japan, the overall SVR12 rate was 100% (165/165), of which HCV in 32% (53/165) of patients harbored A207T, A218S, C316N and Q464E at baseline (Table 2***)***.

**Table 2:** Prevalence of amino acid variants in 165 NS5B genotype 1b Japanese patients at baseline and LDV/SOF treatment outcome in clinical trial.

Amino acid substutition in NS5B region	Prevalence (n/N)	LDV/SOF SVR rate
A207T	33.9 (56/165)	100%
A218S	67.9 (112/165)	100%
C316N	55.8 (92/165)	100%
Q464E	54.5 (90/165)	100%
A207T/A218S/C316N/Q464E	32.1 (53/165)	100%

### Factors Associated with Amino Acid Substitutions in the NS5B Region of HCV Genotype 1b

As shown in Table 1, the demographic and clinical features of the patients were not different between the 50 patients with NS5B-C316 wild-type HCV and the 59 patients with HCV harboring NS5B-C316N. The percentage of patients with previous RBV administration was significantly higher in the former patients than in the latter patients (40.0% vs 20.3%, respectively; *P < *0.05). Consequently, HCV with NS5B-C316N substitution were detected in 61.0% of the patients without previous RBV administration; the ratios were significantly higher than in patients with previous RBV administration (37.5%).

### Locations of Amino Acids Substitutions in NS5B Polymerase of Genotype 1b HCV using Structural Modelling

**Figure 6:**
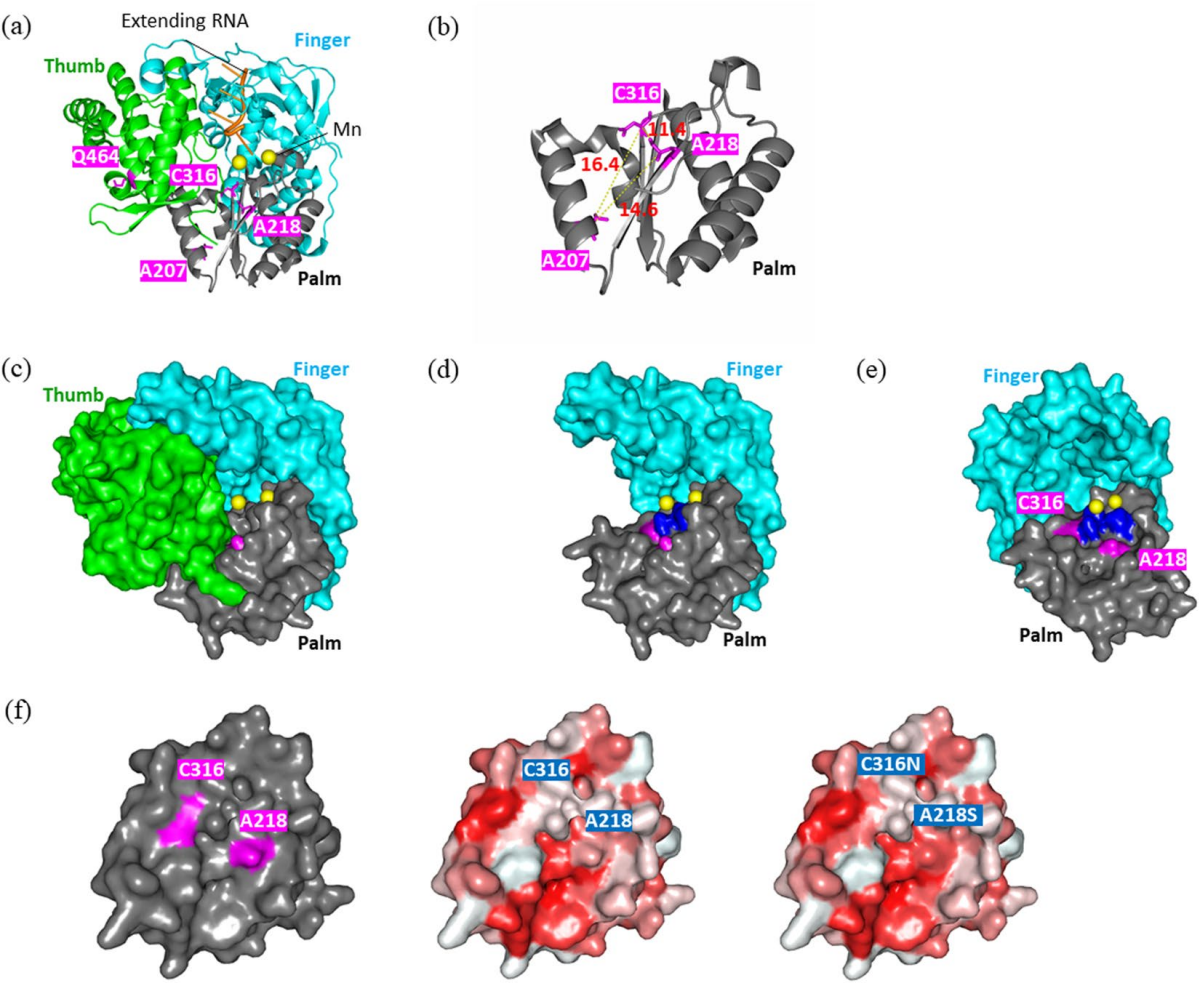
Three-Dimensional Structure of HCV-NS5B Polymerase and Location of the Mutant Amino Acids Seen in HCV Strains Obtained from Patients Showing Virologic Failure after LDV/SOF Therapy. (**a**) Three-dimensional structure of NS5B polymerase as shown using a cartoon model. The amino acids at aa207, aa218, aa316, and aa464 are shown in red. (**b**) Enlarged view of NS5B polymerase showing the distances among aa207, aa218, and aa316. (**c**) Structure of NS5B polymerase in which the surface of each amino acid is visualized. The manganese complexes at the polymerase active site are shown as yellow balls. (**d**) Structure after the deletion of the thumb domain shown in Fig. 2c. (**e**) Structure of NS5B polymerase showing the surface of the NTP tunnel. The NS5B polymerase structure following the deletion of the thumb domain was rotated 45-degrees; aa218 and aa316 are labeled in red, and the activity region consisting of 3 aspartic acids, (aa220, aa318 and aa319) is labeled in blue. (**f**) Circumstances of the NS5B-NTP tunnel with and without A218S and/or C316N mutations. The red and white areas show hydrophobic and hydrophilic characteristics, respectively.

The three-dimensional structure of an NS5B polymerase in HC-J4 strain was shown as a cartoon model in Fig. 6a, in which aa207, aa218, aa316 and aa464 were labeled in red. Among these 4 amino acids, aa207, aa218 and aa316 were located in close proximity to each other in the palm domain. The distances were calculated as 14.6 Å between aa207 and aa218, 11.4 Å between aa218 and aa316, and 16.4 Å between aa316 and aa207 (Fig. 6b), and these amino acids were located along with a nucleotide triphosphate (NTP) tunnel between the palm and finger domains. Thus, in the present study, the locations of aa207, aa218 and aa316 were more precisely examined through the 3-dimensional model showing the surface of each amino acid (Fig. 6c). In this picture, 2 manganese in the active site were shown as yellow balls located at the back of the NTP tunnel. Next, the thumb domain was deleted from the polymerase (Fig. 6d), and the surface of the NTP tunnel in the palm domain was visualized following a 45-degree rotation of the remaining domains (Fig. 6e). As shown in this figure, aa218 and aa316 were located on the surface of the NTP tunnel. The distance between the entrance of the tunnel and the active site was calculated as 22 Å, and activated triphosphate SOF as well as NTPs were shown to link with an extending RNA at a region 10 Å proximal to the active site. Aa218 was exposed on the surface of the NTP tunnel at the upper stream, whereas aa316 was located close to the active site. The NTP tunnel may change depending on the phenotypes of these amino acids; in the wild-type polymerase, C316 and A218 manifested a hydrophilic/basic electrified character and a hydrophobic character, respectively, while both C316N and A218S manifested hydrophilic/non-electrified characters.

### Passage of Activated SOF and UTP in the NTP Tunnel as Evaluated Using Free Energy Analysis Based on Molecular Dynamics Simulations

**Figure 7:**
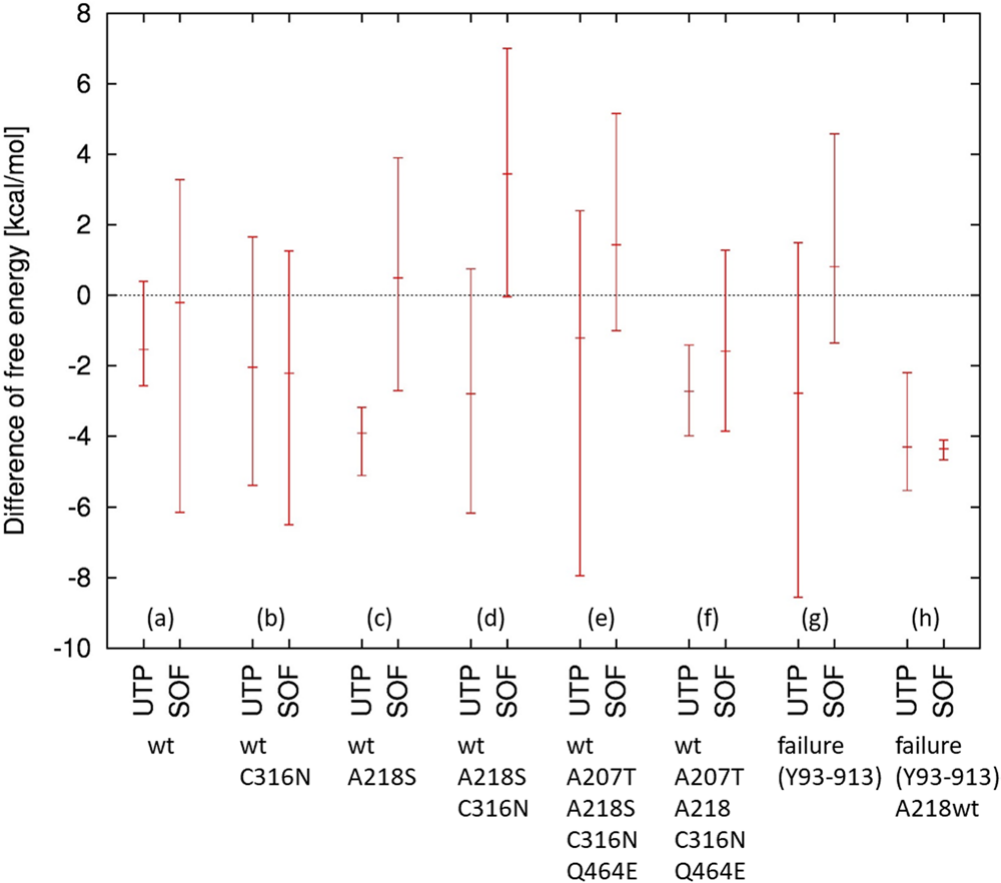
Free Energy Profile Required for the Passage of Uridine Triphosphate (UTP) and Activated Sofosbuvir (SOF) Through the NTP Tunnel of NS5B Polymerase of Genotype 1b HCV. (**a**) NS5B polymerase manifesting the A207, A218, C316 and Q464 wild-types as constructed based on the nucleotide sequence of HCV strains obtained from baseline sera from a patient who achieved an SVR after ledipasvir (LDV)/SOF therapy. (**b**) Single mutation of C316N was added to the polymerase shown in Fig. 3a. (**c**) Single mutation of A218S was added to the polymerase shown in Fig. 3a. (**d**) Double mutations of A218S and C316N were added to the polymerase shown in Fig. 3a. (**e**) Quadruple mutations of A207T, A218S, C316N and Q464E mutations were added to the polymerase shown in Fig. 3a. (**f**) A218S mutation was reverted to A218 wild-type in the polymerase shown in Fig. 3e. (**g**) NS5B polymerase manifesting A207T, A218S, C316N and Q464E mutations constructed based on nucleotide sequences of HCV strains obtained at the time of virologic failure in patients receiving LDV/SOF therapy. (**h**) A218S mutation was reverted to A218 wild-type in the polymerase shown in Fig. 3f. Molecular dynamics simulations were performed 3 times for each situation, and the mean, maximal, and minimal values are show in figure.

Weighted Histogram Analysis Method (WHAM), one of the popular methods for free energy analysis based on molecular dynamics simulations, were performed using 4WTA and 4WTG, in which amino acid residues were substituted by those of the wild-type genotype 1b HCV (HCV-K1-S2) strains and those of the mutant HCV strains in a patient failing to achieve SVR (LC216929). The former polymerase showed an A207/A218/C316/Q464 wild-type phenotype, while the latter polymerase showed an A207T/A218S/C316N/Q464E mutation. In the case with the wild-type NS5B polymerase, the free energies were not different between activated triphosphate SOF and UTP, while the deviation was large in the simulations using activated triphosphate SOF (Fig. 7a). This profile was not changed following the addition of the C316N mutation to the wild**-**type polymerase (Fig. 7b), while the difference became apparent when the A218S mutation was added; the energies required for passage along the NTP tunnel were significantly greater in activated triphosphate SOF than in UTP, suggesting that the therapeutic efficacy of SOF may be deranged in HCV strains carrying the NS5B-A218S mutation (Fig. 7b). A similar difference between activated SOF and UTP was seen when double mutations of A218S and C316N or quadruple mutations of A207T, A218S, C316N and Q464E were added to the wild-type polymerase (Fig. 7c,d), whereas they were absent following the addition of triple mutations of A207T, C316N and Q464E (Fig. 7e). In contrast, in simulations using the mutant**-**type polymerase, the energies required for passage along the NPT tunnel were significantly greater for activated triphosphate SOF than for UTP (Fig. 7f), but this difference disappeared when the A218S mutation was reverted to the A218 wild**-**type (Fig. 7g).

### Genotype 1b Replicon Assay for SOF

The ratios of EC50 values of each mutant HCV strain relative to that of wild-type genotype 1b HCV strain are shown in Table 3. The EC50 fold changes compared to that of a wild-type strain were not different between HCV strains harboring single mutation of A207T, A218S, C316N and Q464E (<2-fold shift in EC50). Moreover, no change in susceptibility was observed for the double mutant (A281S + C316N) and the quadruple mutant (A207T + A218S + C316N + Q464E).

**Table 3:** Genotype 1b HCV Replicon Assay for Sofosbuvir. The ratios of EC_50_ values of the mutant HCV strains relative to that of wild-type HCV strain were shown as mean ± SD.

Amino acid substitution in NS5B region	The Ratios of SOF EC_50_ values relative to that of wild-type HCV
A207T	0.83 ± 0.23
A218S	1.07 ± 0.24
C316N	0.99 ± 0.23
Q464E	0.87 ± 0.17
A218S/C316N	0.85 ± 0.22
A207T/A218S/C316N/Q464E	1.11 ± 0.04

## Discussion

In Japan, virologic failure occurs in some patients with genotype 1b HCV following LDV/SOF therapy in real-world practice. Most patients with virologic failure had a history of previous DCV plus ASV therapies^11,27–29^, and NS5A-RASs such as L31M/V and Y93H substitutions developing at the time of virologic failure during and after DCV + ASV therapy were shown to be responsible for virologic failure after retreatment with LDV/SOF. Also, the HCV strains carrying NS5A-L28M and/or R30Q with Y93H substitutions shows high level of resistance to NS5A inhibitor^5,30^. In our hospital, the amino acid sequences of HCV strains at virologic failure after LDV/SOF therapy were evaluated in 7 patients, and 2 patients had prior DCV + ASV therapy. Various combinations of NS5A-RASs, showing diverse extent of susceptibility to NS5A-inhibitors, were observed, while NS5B-S282T mutation was absent in all the patients. Considering that SOF retains antiviral activity for patients with HCV strains harboring these NS5A-RASs, NS5B amino acid substitutions different from NS5B-S282T mutation might contribute to virologic relapse in patients receiving unsuccessful LDV/SOF therapy.

Amino acid mutations in the NS5B region of genotype 1b HCV were assessed in reference to Con1 and/or HCV-K1-S2 strains, and four variants (A207T, A218S, C316N and Q464E), were detected in all 7 patients experiencing virologic failure after SOF/LDV therapy. For one patient, with available baseline sample, these substitutions were also present at baseline. Across 109 SOF-naïve patients linkage disequilibrium was found among these mutations. The prevalence of patients with HCV harboring all 4 substitutions was 43.1%, and the probability of this result accidentally occurring in 7 consecutive patients was 0.28% (0.431^7^ × 100). This suggests that these substitutions may be enriched among patients failing LDV/SOF treatment, either due to an impact on treatment outcome or due to emergence during therapy due to resistance selection. These observations prompted us to postulate a possible decreased sensitivity of HCV harboring NS5B-A218S substitution to SOF compared to that of wild-type HCV strains. However, no change in susceptibility was observed in the *in-vitro* replicon assay. To further understand this relationship, samples from the 155 genotype 1b patients treated in the LDV/SOF phase 3b clinical study conducted in Japan were assessed. Although the baseline prevalence of these substitutions was similarly high in this group as compared to the other cohort, all patients achieved SVR, further suggesting no impact of these substitutions on treatment outcome. Recently, however, the study group in Kagoshima prefecture showed that NS5B-A218S/N316N mutations were significant factors associated with virologic failure after LDV/SOF therapy by multivariate analysis in about 500 patients with genotype 1b HCV in real-world practice (Personal communication from Dr Seiichi Mawatari and Prof. Akio Ido; Department of Digestive and Life-Style Related Disease, Health Research Course, Human and Environmental Sciences, Kagoshima University Graduate School of Medical and Dental Sciences). Thus, the significance of these substitutions should be further investigated in future.

In phylogenetic tree analysis, HCV strains with NS5B-C316N mutation were located in the leaves different from those in which HCV strains without such mutations were present. The prevalence of patients with genotype 1b HCV harboring the NS5B-C316N substitution have been evaluated in various countries previously, since this mutation is a RAS against dasabuvir, a non-nucleotide type NS5B polymerase inhibitor^31^. In the present study, the prevalence of NS5B-C316N in Japan was 54.1% to 55.8%, and Ito *et al* reported a prevalence of 46.9%^32^, which was similar to the results for our cohort. In contrast, the prevalence were reported to be 95.4% in China^33^, 18.4% in the United States^34^ and 38.1% in Europe^35^. Considering that the prevalence of C316N was lower among patients with previous ribavirin treatment than among ribavirin-naïve patients the trends in antiviral therapies using RBV may determine the frequencies of amino acid mutations in the NS5B region of genotype 1b HCV in each country.

Structural modeling was adopted to evaluate the effect of amino acid substitutions in the NS5B region**:** A207T, A218S and C316N are located in the palm domain, and Q464E are located in the thumb domain. The 3-dimensional structure of a NS5B polymerase in genotype 1b HCV was constructed and the 4 above-mentioned amino acids were labeled. Consequently, aa207, aa218 and aa316 were shown to be located along the NTP tunnel, through which both activated triphosphate SOF and UTP pass. Then, the surface in the palm domain of the NTP tunnel was observed following the deletion of the thumb domain from the 3-dimensional model. As shown in Fig. 6e, aa218 and aa316 were shown to exist on the surface, especially when exposed within the space of the tunnel as in the case of aa218. Thus, the circumstances of the surface were altered depending on the amino acid mutation of aa218 and aa316: from hydrophobic to hydrophilic/non-electrified for the A218S substitution, and from hydrophilic/basic-electrified to hydrophilic/non-electrified for the C316N substitution. A hydroxyl group, showing hydrophilic characteristics, is present at the 2nd-carbon position of the furan ring of UTP, while this position is occupied by a fluorinated-methyl group with hydrophobic characteristics in the case of activated triphosphate SOF. Considering the polarity of amino acids and the furan ring, the passage of activated triphosphate SOF compared with UTP through the NTP tunnel may be deranged in the case of genotype 1b HCV harboring the NS5B-A218S substitution.

The weighted histogram analysis method (WHAM) is one of the most popular method based on molecular dynamics simulations to analyze free energy profile along with a defined reaction coordinate^36^, so we applied this method to estimate the free energy difference required for UTP or activated triphosphate SOF molecules to pass through the tunnel. Although resistance mechanisms in NS5B polymerase against DAAs were evaluated using molecular dynamics simulations^37^, this study sis not focus on the tunnel of NS5B. In the present study, 3-dimensional structures of NS5B polymerase were constructed based on the nucleotide sequences of the genotype 2a JFH-1 strain with modifications as shown in Patients and Methods, since 4WTA/4WTG are currently the only known structures of NS5B polymerase to form complexes with both double-stranded RNA and substrate analogs. The A-chain of 4WTA can become the A-chain of 1NB7 with a C-alpha root-mean square deviation (R.M.S.D) of 2.4 angstroms, and the overall fold is quite similar. Thus, we propose that 4WTA/4WTG structures are appropriate for analyzing the effect of amino acid substitutions on the binding of UTP/SOF to NS5B complexed with RNA. Next, amino acid residues of 4WTA/4WTG were substituted with those of the wild-type genotype 1b HCV (HCV-K1-S2) strains and mutant HCV strains from the serum in other patient with virologic failure after therapy (LC216929). The former polymerase manifested A207, A218, C316 and Q464 wild-type phenotypes, while the latter polymerase carried A207T, A218S, C316N and Q464E substitutions. The movement of activated triphosphate SOF and UTP can be visualized; they pass through the NTP tunnel leading to linkage with an extending RNA at the activity region. The distance of travel was calculated to be about 20 Å. The free energy differences required for activated triphosphate SOF and UTP to pass through the NTP tunnel were evaluated under various situations, and we found that the phenotype of aa218 was a crucial factor determining the difference in energies between activated SOF triphosphate and UTP. Free energy analysis based on molecular dynamics simulations demonstrated that activated triphosphate SOF was inferior to UTP in terms of passage through the UTP tunnel, especially in the case of NS5B polymerase manifesting the A218S substitution, suggesting that the therapeutic efficacy of SOF may be deranged in patients with genotype 1b HCV carrying the NS5B-A218S substitution. NS5B-A218S substitution was less common among patients with previous RBV administration, compared with RBV-naïve patients. Since RBV is an analog of guanine that manifests a hydrophilic character similar to that of UTP, the therapeutic efficacy of SOF may be theoretically improved when RBV is administered together with SOF in patients with HCV strains carrying the NS5B-A218S substitution.

In the present study, we focused on the interaction between activated-SOF and NS5B polymerase, which may be involved in therapeutic efficacy of LDV/SOF therapy in patients with genotype-1b HCV. Thus, deep sequencing analysis was not done in the present study. To clarify the significance of HCV strains carrying NS5B-A218S and C316N mutations, however, deep sequencing analysis should be done especially focusing on dynamics of quasispecies in patients with NS5B-A218S and C316N mutant HCV strains. These matters should be investigated in future.

In conclusion, genotype 1b HCV strains were classified into 2 groups by a phylogenetic analysis based on amino acid sequences of NS5B; HCV strains with and those without NS5B-C316N mutations, which showed high linkage disequilibrium with NS5B-A207T, NS5B-A218S and NS5B-Q464E mutations. The 7 patients experiencing virologic failure harbored these 4 substitutions. Structural modeling and free energy analyses revealed that the NS5B-A218S substitution may have an effect on the passage of activated triphosphate SOF through the NTP tunnel. Overall, the presence of these substitutions individually or in combination with NS5A-RASs did not impact the treatment outcome to LDV/SOF, however, further studies are needed to elucidate possible effects in patients with advanced disease.

## Patients and Methods

### Patients and Blood Samples

Three patient groups were assessed. The first group included 6 patients who were referred to Saitama Medical University Hospital after experiencing virologic relapse to LDV/SOF. The second group included 109 SOF-naïve patients seen at our hospital between September 2015 and September 2016. 92 of whom received LDV/SOF, and 17 of whom received ombitasvir (OBV), paritaprevir (PTV) with booster ritonavir (r). Baseline serum samples were obtained from all 109 patients and serum samples were also obtained at the time of virologic failure for the 6 patients in the first group and for the 1 subject who failed treatment with LDV/SOF in the second group. The third group included165 Japanese patients with genotype 1b chronic HCV infection enrolled in a Phase 3b clinical trial in Japan evaluating LDV/SOF (ClinicalTrails.gov identifier: NCT01975675).

Written informed consent was obtained from all the patients prior to the collection of blood samples. The study conformed to the ethical guidelines of the Declaration of Helsinki and was conducted with the approval of the Institutional Review Board of Saitama Medical University Hospital.

### Evaluation of Virologic Characteristics

HCV-RNA was purified from sera and subjected to nested PCR followed by direct sequencing. The amino acid substitutions were assessed by referring to the sequences of the Con1 strain (GenBank accession number AJ238799.1) and the HCV-K1-S2 strain (D50485.1). HCV strains with or without mutations were classified according to a phylogenetic tree analysis based on amino acid sequences in the NS5B regions. Also, the 3-dimensional structure of NS5B polymerase was evaluated using a bioinformatics analysis, and the significance of the mutations was assessed using molecular dynamics. Furthermore, the ratios of EC50 values of each mutant HCV strain for SOF relative to that of wild-type genotype 1b HCV strain were evaluated using genotype 1b HCV replicon assay.

#### Direct Sequencing of the NS5A and the NS5B Region of HCV

HCV-RNA was purified from sera using the QIAamp MinElute Virus Spin Kit (Qiagen K.K., Tokyo, Japan) and was subjected to nested PCR followed by direct sequencing of NS5A and NS5B regions. Reverse transcription and the first PCR amplification were performed for purified RNA samples using the PrimeScript™ II High Fidelity One Step RT-PCR Kit (TaKaRa Bio Inc., Seta, Japan) with primer sets: set of NS5A_L/NS5A_R for aa12–125 in the NS5A region, and HCV-16L/HCV-19R for full-length NS5B region (Table 4). For direct sequencing of the NS5B region, a second PCR was performed using Tks Gflex™ DNA Polymerase (TaKaRa Bio Inc.) with 3 sets of primers: HCV-16.5 L/HCV-17R, HCV-18L/HCV-18R, and HCV-19L/HCV-18.5 R. Each fragment was purified using the QIAquick PCR Purification Kit (Qiagen K.K.) or the QIAquick Gel Extraction Kit (Qiagen K.K.) and was sequenced using the BigDye^®^ Terminator v3.1 Cycle Sequence Kit (Applied Biosystems, CA, US) using the PCR primers. Direct sequencing was performed using a 3730xl DNA Analyzer (Applied Biosystems), and the resulting nucleotide sequences were assembled using ATGC for Windows version 8 software (GENETTX, Corp., Tokyo, Japan). The nucleotide mixture detection during sequencing was determined automatically by the software. All working processes were performed according to the manufacturer’s protocol.

**Table 4:** A Set of Primers Used for RT-PCR and Direct Sequencing in NS5A and NS5B region.

Name	Nucleoside Sequences (5′ → 3′)	Nt Position*	
***NS5A region***			
NS5A_L	AGGGATGTTTGGGACTGG	6273–6290	1st PCR, sense
NS5A_R	CCGTCACGTAGTGGAAATC	6633–6651	RT, 1st PCR, anti-sense
***NS5B region***			
HCV-16L	GTCCTGGAAGGACCCGGACTACGTC	7238–7262	1st PCR, sense
HCV-16.5 L	AGCTGGTGAGGACGTCGTCTGCTGC	7574–7598	2nd PCR, sense
HCV-17L	GCAGAAGAAGGTCACCTTTGACAGA	7742–7766	2nd PCR, sense
HCV-18L	GGTGAATACCTGGAAATCAAAGAAA	8210–8234	2nd PCR, sense
HCV-19L	CTCGCACGGGCTGCGTGGGAGACAG	8772–8796	2nd PCR, sense
HCV-16R	TCATCTCCTTGAGCACGTCCCGGTA	7788–7812	2nd PCR anti-sense
HCV-17R	TCGGGGGCCAAGTCACAACATTGGT	8317–8341	2nd PCR, anti-sense
HCV-18R	AGAAATGAGTCATCAGAATCATCCT	8862–8886	2nd PCR, anti-sense
HCV-18.5 R	TGGCCTGGAGTGGTTAGCTCCCCGT	9375–9399	2nd PCR, anti-sense
HCV-19R	AGGGAATGGCCTATTGGCCTGGAGT	9389–9413	RT, 1st PCR, anti-sense

*nucleotide position according to Con1 (AJ238799).

The amino acid sequences in the NS5A and NS5B regions of the HCV strains were determined using GENETYX for Windows version 13 (GENETYX Corp.), and the mutations were assessed by referring to the sequences of the Con1 strain (GenBank accession number AJ238799.1) and the HCV-K1-S2 strain (D50485.1). HCV strains with or without mutations were classified according to a phylogenetic tree analysis based on amino acid sequences in the NS5B regions. Also, the 3-dimensional structure of NS5B polymerase was evaluated using a bioinformatics analysis, and the significance of the mutations was assessed using molecular dynamics.

The nucleotide sequences of the HCV strains in patients before LDV/SOF therapies and in those at viral relapse after the therapies were submitted to the DDBJ/EMBL/GenBank databases under accession numbers LC210145 to LC210252 and LC216928 to LC216934, respectively.

For the patients in the LDV/SOF clinical trial, the HCV NS5B coding regions were amplified by DDL Diagnostic Laboratory (Rijswijk, Netherlands) using standard reverse transcription polymerase chain reaction (RT PCR) technology, in available plasma/serum samples with HCV RNA was > 1000 IU/mL. Deep sequencing using MiSeq platform (Illumina, Inc., San Diego, CA) was performed by DDL or WuXi AppTec (Shanghai, China).

#### Construction of Phylogenetic Trees

The amino acid sequences of the NS5B regions obtained by direct sequencing were multiple-aligned using MEGA7: Molecular Evolutionary Genetics Analysis version 7.0 for bigger datasets, and the phylogenetic trees were constructed using the maximum likelihood method adapted with best fit model (Kimura 2-parameter plus Gamma distributed with Invariant sites). To confirm the reliability of the phylogenetic analysis, bootstrap resampling was performed 1,000 times.

#### Structural Bioinformatics Modeling of HCV NS5B Polymerase

The three-dimensional structure of NS5B polymerase in genotype 1b HCV was constructed based on nucleotide sequences of the HC-J4 strain (1NB7) using the software PyMOLTM 1.8.x (http://pymol.org). Mutant amino acids were labeled as various colors and their locations were evaluated using a cartoon model or a model showing the surface of each amino acid.

#### Free Energy Analysis for HCV NS5B Polymerase and Activated Triphosphate SOF and Uridine Triphosphate

The model structures of NS5B polymerase for the molecular dynamics simulations were generated based on the crystal structures with PDB ID of 4WTA (with uridine diphosphate (UDP)) and 4WTG (with activated triphosphate SOF) substituting the amino acid residues using SCWRL 4^38,39^. The system was solvated with TIP3P water molecules and chloride ions using the LEaP module of AmberTools version 1.5^40^, relaxed and sampled for more than 3 ns in total before starting the WHAM simulations.

All the simulations were executed using GROMACS ver.4.6.5^41^ with the AMBER ff14SB force field^42^ for proteins and parameters generated by R.E.D. Server^36^ for UTP and activated triphosphate SOF. To determine the potential of the mean force (PMF) of UTP/SOF through the tunnel, the weighted histogram analysis method (WHAM)^42^ with 25 2-ns windows (50 ns in total) was used. This method is one of the popular methods to obtain free energy profile along a defined reaction coordinate. The distance from the O3 atom in U3 (chain ID: P) and the C5 atom in UTP/SOF was chosen as the reaction coordinate (10–22 Å).

Each of the PMF simulations was repeated 3 times per model. The models subjected to the analysis were as follows, 4WTA and 4WTG in which amino acid residues were substituted with those of the wild-type genotype 1b HCV (HCV-K1-S2) strains, 4WTA and 4WTG adding either of C316N, A218S, A218S/C316N, A207T/C316N/Q464E or A207T/A218S/C316N/Q464E substitutions to the wild-type HCV strain, 4WTA and 4WTG substituted with amino acid residues of the mutant type genotype 1b HCV strain obtained from a patients failing to achieve SVR (LC216929) and 4WTA and 4WTG in which an amino acid residue at aa218 was reverted from A218S mutation to A218 wild-type in the mutant HCV strain.

#### Genotype 1b HCV Replicon Assay for SOF

NS5B substitutions were introduced into the genotype 1b replicon by site-directed mutagenesis and tested in transient transfections as previously described^43^. Replicon RNAs were transcribed *in vitro* from replicon-encoding plasmids using a MEGAscript kit (Ambion, Austin, TX). RNA was transfected into Huh-lunet cells using the method of Lohmann *et al* ^44^. Compound were diluted in 100 percent DMSO and added to cells. Cells were treated for 3 days, after which culture media were removed, cells were lysed, and Renilla luciferase activity was quantified using a commercially available assay (Promega, Madison, WI) and a Top Count instrument (Perkin Elmer, Waltham, MA). EC50 values were calculated as the compound concentration at which a 50 percent reduction in the level of Renilla reporter activity was observed when compared with control samples with DMSO.

#### Statistical Analysis

Categorical data were compared using the Fisher exact test. Distributions of continuous variables were analyzed using the Mann-Whitney U-test to identify factors associated with NS5B-RAS. All the tests of significance were two-tailed, and P values of less than 0.05 were considered statistically significant. SPSS Statistics version 22 (IBM SPSS, Tokyo, Japan) was used for the analyses.
